# Components of Behavioral Activation Therapy for Depression Engage Specific Reinforcement Learning Mechanisms in a Pilot Study

**DOI:** 10.5334/cpsy.81

**Published:** 2022-10-13

**Authors:** Quentin J. M. Huys, Evan M. Russek, George Abitante, Thorsten Kahnt, Jacqueline K. Gollan

**Affiliations:** 1 Division of Psychiatry, University College London, London, UK; 2 Max Planck UCL Centre for Computational Psychiatry and Ageing Research and Wellcome Trust Centre for Human Neuroimaging, Institute of Neurology, University College London, London, UK; 3 Camden and Islington NHS Foundation Trust, London, UK; 4 Department of Psychiatry and Behavioral Sciences, Northwestern University Feinberg School of Medicine, Chicago, IL, USA; 5 National Institute on Drug Abuse Intramural Research Program, Baltimore, MD, USA; 6 Department of Neurology, Northwestern University Feinberg School of Medicine, Chicago, IL, USA; 7 Department of Psychiatry and Behavioral Science, Northwestern University Feinberg School of Medicine, Chicago, IL, USA

**Keywords:** decision-making, learning, Pavlovian reflexes, instrumental learning, Orthogonalized Go/No learning, Behavioral Activation treatment, reward learning

## Abstract

**Background:**

Behavioral activation is an evidence-based treatment for depression. Theoretical considerations suggest that treatment response depends on reinforcement learning mechanisms. However, which reinforcement learning mechanisms are engaged by and mediate the therapeutic effect of behavioral activation remains only partially understood, and there are no procedures to measure such mechanisms.

**Objective:**

To perform a pilot study to examine whether reinforcement learning processes measured through tasks or self-report are related to treatment response to behavioral activation.

**Method:**

The pilot study enrolled 13 outpatients (12 completers) with major depressive disorder, from July of 2018 through February of 2019, into a nine-week trial with BA. Psychiatric evaluations, decision-making tests and self-reported reward experience and anticipations were acquired before, during and after the treatment. Task and self-report data were analysed by using reinforcement-learning models. Inferred parameters were related to measures of depression severity through linear mixed effects models.

**Results:**

Treatment effects during different phases of the therapy were captured by specific decision-making processes in the task. During the weeks focusing on the active pursuit of reward, treatment effects were more pronounced amongst those individuals who showed an increase in Pavlovian appetitive influence. During the weeks focusing on the avoidance of punishments, treatment responses were more pronounced in those individuals who showed an increase in Pavlovian avoidance. Self-reported anticipation of reinforcement changed according to formal RL rules. Individual differences in the extent to which learning followed RL rules related to changes in anhedonia.

**Conclusions:**

In this pilot study both task- and self-report-derived measures of reinforcement learning captured individual differences in treatment response to behavioral activation. Appetitive and aversive Pavlovian reflexive processes appeared to be modulated by separate psychotherapeutic interventions, and the modulation strength covaried with response to specific interventions. Self-reported changes in reinforcement expectations are also related to treatment response.

**Trial Registry Name:**

Set Your Goal: Engaging in GO/No-Go Active Learning, #NCT03538535, http://www.clinicaltrials.gov.

## 1 Introduction

Depression is a common illness with a heavy toll on societies across the globe (Whiteford et al., 2013). Although many treatment strategies exist, a substantial number of individuals respond poorly or not at all (e.g. Rush et al. 2006). One problem is that it has been difficult to establish differential predictors of likely treatment response, both for pharmacological and psychotherapeutic approaches (Webb et al., 2019; Schmaal et al., 2015; DeRubeis et al., 2014; McGrath et al., 2013; DeBattista et al., 2011; Schweizer et al., 2020). One solution may lie in the identification of the targets of treatments and the use of these treatment targets for treatment allocation (Paulus et al., 2016). However, it has been challenging to identify specific relationships between therapy techniques and changes in behaviour or cognitions (Arch et al., 2012; Longmore and Worrell, 2007).

A specific approach with some theoretical promise is the study of specific psychotherapeutic treatment components with cognitive computational methods such as reinforcement learning (Huys et al. 2016; Reiter et al., 2021). Reinforcement learning is a field at the intersection of machine learning and neuroscience which has undergone substantial development over the past three decades. Reinforcement learning theories have provided fundamental insights into how individuals learn from, optimize and behave in the face of rewards (Daw et al., 2005; Daw and Dayan, 2014; Huys et al., 2015; Russek et al., 2017; Mattar and Daw, 2018; Liu et al., 2021), and how this relates to neural circuits (Schultz et al., 1997; Daw et al., 2011; Eshel et al., 2015), mood (Rutledge et al., 2015, 2017), depression (Kumar et al., 2008; Huys et al., 2013; 2016; Geugies et al., 2019), and treatment response and long-term course of depression (Berwian et al., 2020).

However, what has been relatively less explored is whether and how reinforcement learning could help in understanding and improving psychotherapeutic approaches. Indeed, there appears to be only one recent study, which suggests that impairments in reinforcement learning are associated with depression; and altered by cognitive behavioural therapy (Brown et al., 2021). This is particularly surprising because reinforcement learning and some therapeutic approaches like behavioural activation (BA)—an evidence-based psychological treatment (Dimidjian et al., 2006; 2017; Dichter et al., 2010; Martell et al., 2010)—have a shared focus on how behavior is motivated by reinforcements, and indeed share some foundational literature (Lewinsohn et al., 1979; Meehl, 1975; Beck, 1976). Like other treatments, however, BA may have no beneficial effect for up to one-third of patients: meta-analyses show that BA has a moderate treatment effect (d = 0.34) and a remission rate (56% vs 30%) compared to treatment as usual (Dimidjian et al., 2017). This modest efficacy rate underscores the need to optimize patient learning in psychological treatments to restore psychiatric function. Indeed, impaired reward learning and loss of pleasure in formerly enjoyable activities (anhedonia) are associated with poorer treatment outcomes. Reduced reward learning predicts greater odds of a persisting MDD diagnosis post-treatment (OR = 7.84) and anhedonia predicts non-response (OR = 6.00) and non-remission (OR = 9.28) among adult inpatients with MDD (Vrieze et al., 2013; 2014). One interesting potential route is through the application of formal reinforcement learning theories. A number of studies have sought to test the component mechanisms through which BA could facilitate recovery, including energizing the patient to seek and remember pre-existing or new reward experiences, and reducing the patient’s avoidance of depressogenic/aversive situations, and there is evidence that reward mechanisms may be involved in mediating the BA treatment effect (Kalsi et al., 2017; Nagy et al., 2020). However, BA predates the extensive formal development of RL theory in neuroscience and as such the RL foundations of BA have not yet been tested formally.

BA learning theory emphasizes a positive feedback loop whereby negative mood reduces behaviors necessary to gain access to rewarding experiences, and the low reinforcement or excessive punishment contingent on this then further lowers mood (Martell et al., 2010). Indeed, the American Psychological Association recommends monitoring the reduction of avoidance and increase of reward experience while using Behavioral Activation psychotherapy for major depression as interference of these two dimensions may limit recovery. At its core, the BA model hence relies on a link between reward expectation and activation, and loss or low reward expectations and inactivity. Such links are readily apparent in animal and human Pavlovian decision-making. For instance, Guitart-Masip et al. (2012) used a task in which individuals had to learn whether to emit an active go action, or withhold the active go action to obtain rewards. While participants were able to learn the active response to obtain rewards, they were profoundly impaired when they were required to inhibit action to obtain rewards. A similar, but opposite pattern is observable in the aversive domain, where negative expectations promote inhibition. This has been formally traced to the influence of appetitive and aversive Pavlovian influences, respectively.

Here, we aimed to perform a pilot study to test the RL mechanisms underlying BA therapy response formally, taking a dual approach. First, we repeatedly administered the orthogonalized Go/No-Go Learning Task to examine whether BA alters Pavlovian processes, and whether these relate to specific parts of the BA therapy. Second, the key measure of BA efficacy are changes in self- or observer-reported depression severity symptoms. In principle, the theory underlying BA thereby suggests that self-reported symptoms should follow the rules of reinforcement rules. To our knowledge, this has never been studied. We aim to address it by examining how self-reports about reinforcement experience and expectations change over the course of therapy, and whether these satisfy the formal processes stipulated by RL theories.

Specifically, we anticipated that components of the therapy emphasizing active engagement with rewards would be paralleled by an *increase* in appetitive Pavlovian influence on choice in those individuals who respond to the intervention. Similarly, we anticipated that components of the therapy emphasizing active engagement despite aversive expectations should be paralleled by a *reduction* in aversive Pavlovian inhibition on choice in those individuals who respond to the intervention. Finally, we hypothesized that self-reported reinforcement experiences and anticipation would follow formal RL theory predictions, and that the learning rate in self-reported anticipations would relate to symptomatic response to BA therapy.

## 2 Methods

### 2.1 Study population

**Figure 1:**
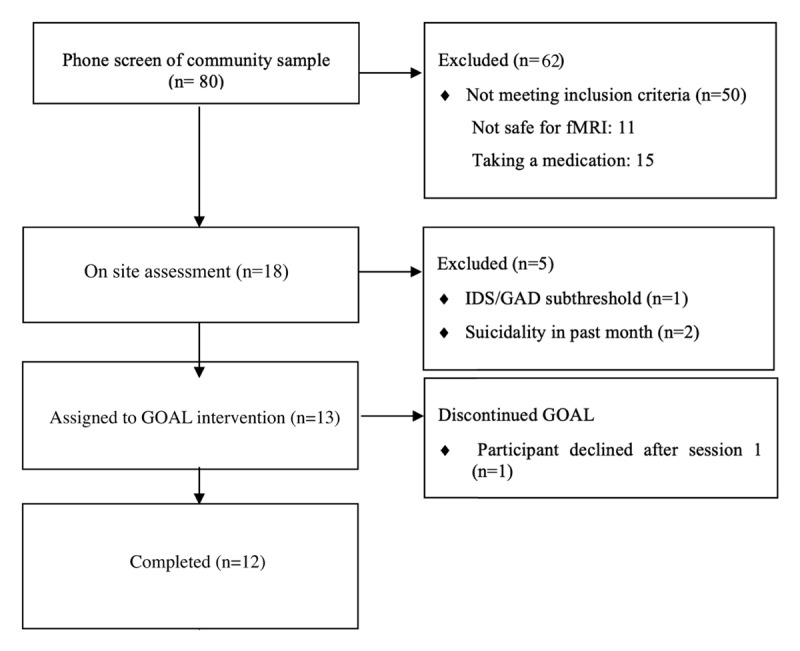
CONSORT (Consolidated Standards of Reporting Trials) diagram.

We recruited participants from the city and suburbs surrounding Northwestern Memorial Hospital, an academic medical center in Chicago, Illinois. Of the 80 people screened by phone, 18 (22.5%) were invited for on-site baseline assessment. Once these 18 participants evaluated on-site, 13 met criteria to enroll during the baseline assessment and were assigned to the BA intervention. Of the 13 who were assigned (our intent to treat group), 12 completed the intervention and evaluations. See CONSORT chart (Figure 1).

We enrolled male and female adults between ages 21 years and 45 years with scores >24 on the Inventory of Depressive Symptomatology, Self-Report (IDS-SR; Rush et al. 2000) and depression diagnoses on the Mini-International Neuropsychiatric Interview (MINI 7.0.2; Sheehan et al. 1998). Participants reported comorbid psychiatric conditions including bipolar disorder, suicidality, and substance abuse. Subjects reported that they were medically healthy.

### 2.2 Procedure

**Figure 2:**
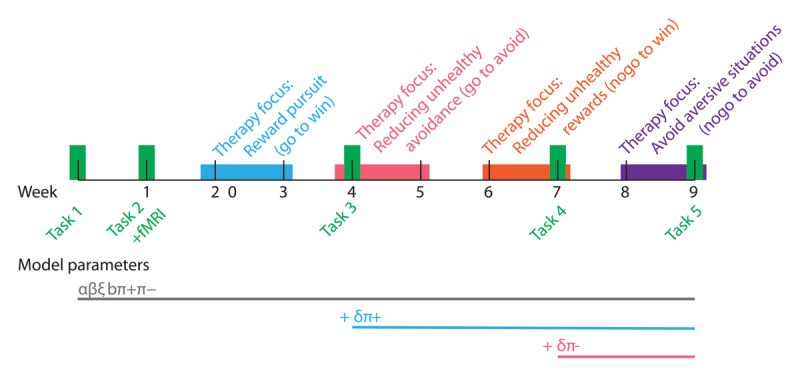
Timeline. The therapeutic interventions are shown in color. The green bars indicate the timing of the task administrations. The bar below shows the model parameters. From task administration 3 onwards, an additional appetitive Pavlovian bias was included, and from administration 4 onwards an additional aversive Pavlovian bias.

The overall timeline is shown in Figure 2. We screened prospective volunteers by phone to discern eligibility before coming to the lab. In the first visit to the lab, we conducted a MINI-based clinical interview to assess type, severity, and timing of lifetime psychiatric symptoms. One week later, at the second visit in the lab, we completed the Orthogonalized Go/No-Go task. After these evaluations, participants started the nine-week BA treatment program. A key part of the BA intervention is the planning and execution of activities. Each day during the intervention (n = 56 days) participants performed at least one activity and planned an activity for the next day and they filled out the Go/No-Go Active Learning (GOAL) form. Specifically, participants were asked to plan and perform the following types of activities:

- Emotional activities that aim to accept or experience your feelings
- Mental activities that challenge yourself to think about new ideas
- Physical activities that improve your physical health
- Pleasure activities that increase your joy or delight
- Sensory activities that increase your sight, smell, sound, taste or touch
- Social activities connecting with others
- Spiritual activities that show your values

Thereafter, participants were repeatedly evaluated for depression each week for depression using the IDS-SR. The Go/No-Go task was performed five times: at weeks 0, 1, 4, 7 and 9. The timing of the task administrations was chosen to approximately match the intervention while attempting to minimize participant burden.

### 2.3 Behavioral Activation treatment

We used a participant manual to guide BA training. We clustered the BA treatment strategies to focus on two functions: The first goal was to teach the patient how to detect, use, and remember to seek rewards (e.g., identifying, scheduling, completing daily goals of enjoyment and mastery) while also detecting and stopping excessive reward seeking (e.g., identify the ‘true’ value of rewarding choices to reduce reward overconsumption). The second goal was to teach the patient to use avoidance in harmful, not challenging, situations (e.g., physically move away from genuinely aversive situations or when having depressogenic ruminations), while reducing avoidance when skills and habituation could be achieved. The treatment schedule of nine weekly 50-minute sessions delivered by a clinical psychologist (JKG) was based on prior research.

### 2.4 Orthogonalized Go/No-go task

**Figure 3:**
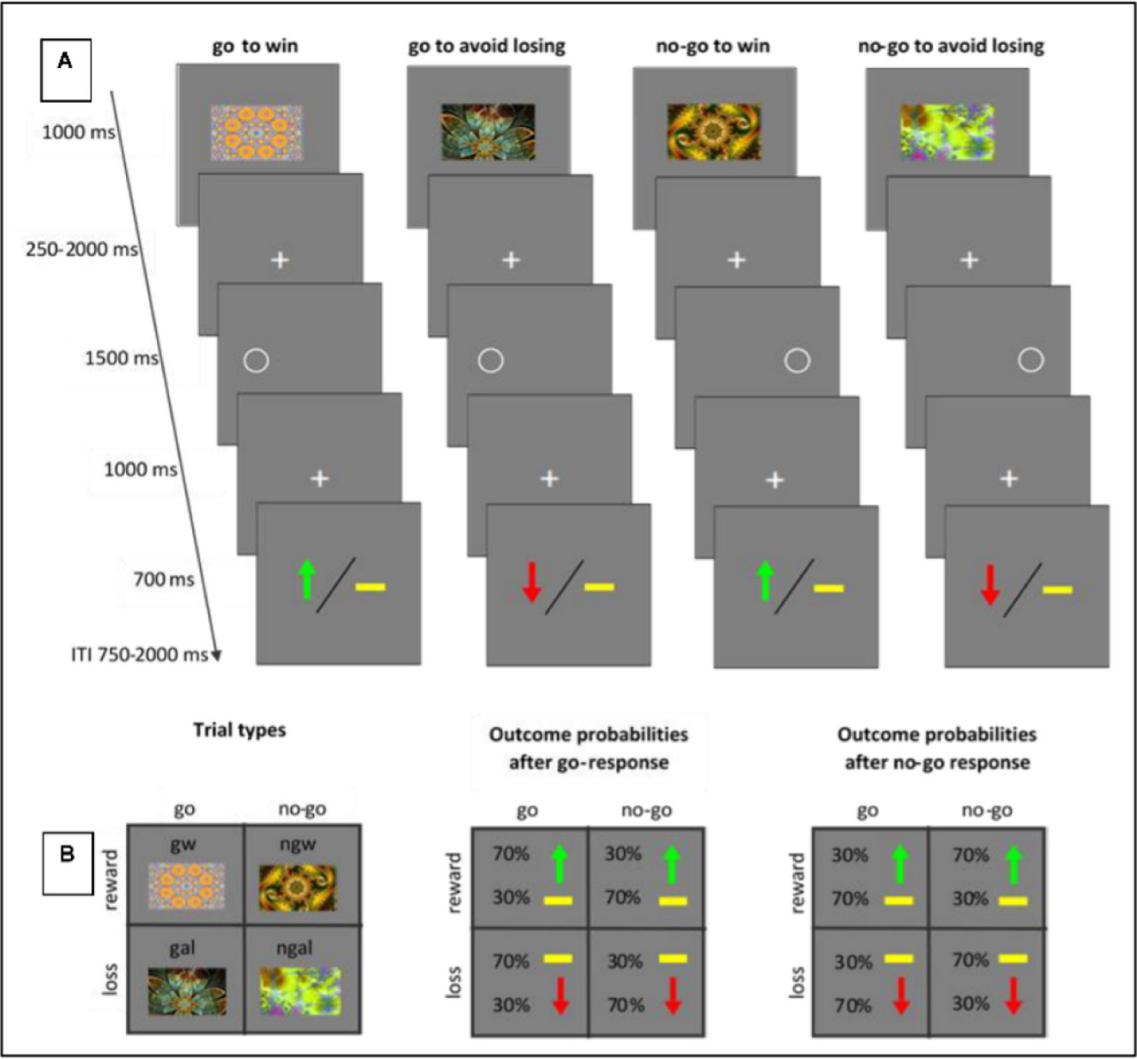
Experimental paradigm for Orthogonalized Go/No-go task. **A)** On each trial, subjects see one of four fractals. After a fixation period, a target is shown an subjects have to either go and respond to the target with the correct key (left for left stimulus, right for right stimulus), or nogo. There are 20 practice trials for target detection, followed by 240 trials (60 trials per condition), divided into nine minute sessions. Completion takes 36 minutes. Total amount that can be earned is USD 24. **B)** There are four trial types, and the probability of the outcomes are shown for both a go- and a nogo response for each trial type.

The Orthogonalized Go/No-go task (Figure 3) used a balanced 2(win/loss) and 2(go/no-go) factorial design to generate four trial conditions. Each condition had a distinct visual stimulus (fractal image). Participants viewed the stimulus, and had 1.5s to choose whether to emit an active response (go) or not (no-go). They were then informed of the outcome, which was probabilistic (80%/20%). Participants learned through the reward and loss feedback whether to emit a go or nogo action for each stimulus. For instance, for the go-to-win (Figure 2) stimulus, if participants performed a go action, they would observe a reward on 80% of the trials, and no reward on 20% of the trials. If they performed a no-go action, they would observe a loss on 80% of the trials, and a reward on the other 20%. Conversely, for a nogo-to-avoid-loss stimulus (Figure 2), if participants performed a nogo, they observed no loss (avoided the loss) on 80% of the trials, and a loss on 20% of the trials, and if they performed a go, they observed a loss on 80% of the trials and no loss on 20%.

### 2.5 GOAL form

The GOAL form is a new questionnaire designed to elicit key variables of RPE learning. The GOAL form asks participants to rate the activities they scheduled and performed in terms of a) their expectation of how rewarding or punishing the activities scheduled for the next day will be; b) how rewarding or punishing the performed activities on that day have been; c) how rewarding or punishing they expect the performed activities to be in the future. By comparing either of the two expectations with the actually experienced reinforcement, a type of prediction error can be computed.

### 2.6 Analytic Methods

Descriptive statistics are used to describe the sample, percentages for discrete characteristics, and measures of central tendency for continuous characteristics. Effect size was calculated to assess treatment effect.

Behavioral responses in the task were first subjected to a traditional analysis. Learning accuracy was tested using a three-way ANOVA with time (10 trials in each bin), the action (go/no-go) and valence (win/lose) as repeated factors. This was repeated for sensitivity to value (reward/loss). Behavioral learning was defined as: (a) Pavlovian bias = percent accuracy of (1) Pavlovian (go to win) bias – instrumental (no-go to win) learning; and (2) Pavlovian (no-go to avoid) bias – instrumental (go to avoid) learning); (b) Learning rate for wins and losses; that is, how long it takes the participant to select (learn) the correct action, which is defined as the percent time between the different fractal images and task expectations; (c) Reaction time = response speed in ‘go’ trials determined by the data on the button press reaction times to targets, along with the proportion of trials in which button press reaction times exceed the response deadline; (d) Sensitivity to value = ability to use the win/loss feedback to guide their learning during the task (the value assigned to the expected outcome).

### 2.7 Computational modelling of task data

The behavioral responses in the go/no-go task were then subjected to computational modelling to quantify the underlying learning processes. For the baseline, we followed the approach previously employed (Guitart-Masip et al. 2012). At baseline, we built and compared models to answer the extent to which: (1) there is a Pavlovian component; (2) this Pavlovian component differs in the reward and loss domains. To achieve this, we fitted a series of models in which the probability to emit one of the two available actions *a* (go or nogo) when faced with stimulus *s* was:


1 \[p(a,s) = \frac{{{e^{{\cal Q}(a,s)}}}}{{\sum\nolimits_{a^{\prime}} {{e^{{\cal Q}(a^{\prime},s)}}} }}\]

The models differed in how the Q value was constructed. In the simplest model, the Q value captured instrumental learning only according to Rescorla-Wagner learning:


2 \[{\cal Q}_t^{{\mathrm{RW}}}(a,s) = {\cal Q}_{t - 1}^{{\mathrm{RW}}}(a,s)\,\, + \alpha (\beta {r_t} - {\cal Q}_{t - 1}^{{\mathrm{RW}}}(a,s))\]

The next model additionally allowed for an irreducible noise term, such that the decision probability became:


3 \[p(a,s) = \left[ {\frac{{{e^{{\cal Q}(a,s)}}}}{{\sum\nolimits_{a^{\prime}} {{e^{{\cal Q}(a^{\prime},s)}}} }}} \right](1 - \xi) + \frac{\xi }{2}\]

We then allowed for an additional bias term, which was a temporally fixed parameter *b* added to the \[{\cal Q}\] value for the go action. Finally, we added a Pavlovian component. This assumed that in addition to the instrumental \[{\cal Q}\] value participants also learned a Pavlovian value \[{\cal V}\]:


4 \[{{\cal V}_t}(s) = {{\cal V}_{t - 1}}(s) + \alpha (\beta {r_t} - {{\cal V}_{t - 1}}(s))\]

This value does not depend on the action, but only on the stimulus, such that the two stimuli leading to rewards or no outcome assumed an overall positive Pavlovian value, while the two stimuli leading to losses or no outcomes assumed an overall negative Pavlovian value. The \[{\cal Q}\] value for this model was then:


5 \[
{\cal Q}_t^{{\mathrm{Pav}}}(a,s) = {\begin{cases}
\quad{{\mathrm{if}} \ a\ {\mathrm{ is\ go}}: \ {\cal Q}_t^{{\mathrm{RW}}}(s,a)\,{\mathrm{ + }}\pi {\cal V}({\mathrm{s}})}\\
{{\mathrm{if}}\ a \ {\mathrm{ is\ nogo}}:\ {\cal Q}_t^{{\mathrm{RW}}}(s,a)}
\end{cases}}
\]

Finally, the model with two Pavlovian parameters allowed for a different Pavlovian parameter *π* for the appetitive and aversive stimuli. To capture the effect of the therapy on decision-making in the task, we assumed that all parameters were fixed for each subject over the different sessions, except for the appetitive and aversive Pavlovian component. The appetitive Pavlovian component was allowed to freely vary from task administartion 3 onwards (session 4, c.f. Figure 2), whereas the aversive Pavlovian component was allowed to change from task administration 4 onwards (session 7).

Group-level models were fitted using hierarchical Bayesian formulations and expectation-maximization estimation schemes, which also provide the necessary information for model comparison. Bayesian model comparison was employed to identify the model that most parsimoniously explains the data, i.e., capture performance data without overfitting it. Parameters were extracted from the most parsimonious model only.

### 2.8 Computational modelling of GOAL form data

The responses of the GOAL form were subjected to modelling in order to determine whether reward predictions, punishment predictions and choices are consistent with formal reinforcement learning theory, and whether the extent of these processes relate to improvements in anhedonia symptoms. For each activity completed, we computed reward prediction errors as the reward difference between reward reported upon completing the activity and reward predicted when the activity was planned, and punishment prediction errors analogously. In order to determine whether reward prediction errors drive immediate changes in reward predictions (Figure 6a), we computed change in reward prediction as the difference between reward predicted when the activity is planned and reward predicted immediately after it is completed. In order to determine whether reward prediction errors drive immediate changes in reward predictions over longer time scales (Figure 6c), we computed a second measure of reward prediction change as the difference between reward predictions for successive times the same activity is planned. For both of these analysis, we examined whether there was an effect of reward prediction error on reward prediction change by fitting a linear mixed effect model predicting reward prediction change as a function of reward prediction error. For both of these, we performed the same analysis, however using punishment predictions and reported punishments in place of reward predictions and reported rewards (Figure 6b,d).

In order to examine whether previous rewards and punishments drive choices (Figure 6e,f) we fit a generalized linear mixed effects model (mixed effects logistic regression) to predict whether an activity would be repeated the next day as a function of the reward and punishment reported on the prior day.

In order to examine whether the effect of reward prediction errors and punishment prediction errors on reward prediction change and punishment prediction change was greater in individuals that have larger improvements in anhedonia symptoms (IDS-SR items 21 or 19), we fit a linear mixed effects model to predict reward prediction change as a function of reward prediction error, the change in either 21 or 19 between week 2 (when therapy started) and the end of the experiment, and their interaction. We chose to focus on specific items of the IDS-SR questionnaire as we hypothesized that reward-related learning should be most directly relevant to reward-related symptoms, and less directly or immediately related to other symptoms of the broader depression syndrome captured by the IDS-SR. However, we note that these items have not been validated independently.

All mixed effects models were fit, and p-values computed, using the MixedModels.jl package in the Julia programming language (Bates et al., 2021). For all models, all regressors, except for the change in IDS-SR measures (for which there was only one measure per participant) were entered as both fixed effects and random (per subject) effects. For analysis that examined interactions with IDS-SR items 19 and 21, p-values were Bonferroni corrected for two hypotheses (one for either item).

## 3 Results

### 3.1 Pretreatment characteristics

Most subjects were diagnosed with current depression (90.9%) of moderate severity (mean IDS-SR =24.5, SD = 8.5). See Table 1.

**Table 1:** Clinical variables.


DIAGNOSES		FREQUENCY N (%)

MINI	MDD current	13 (100%)

	MDD past	8 (73%)

	Bipolar II current	1 (9%)

	Anxiety comorbidity	5 (46%)

**SCALES**		**MEAN (ST. DEV.)**

IDS-SR		34.5 (8.5)

GAD-7		9.9 (3.9)

STAI	State	43.9 (6.9)

	Trait	54.3 (7.6)


### 3.2 Treatment response

**Figure 4:**
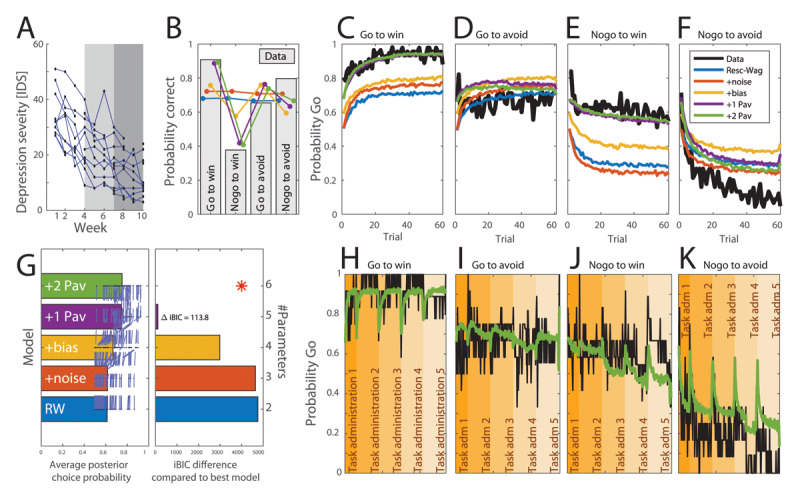
Computational modelling of task data. **A:** Individual trajectories of depression severity measurements. The grey areas show the three periods of therapy between different task administrations. **B:** Average probability correct in each of the four task conditions (gray bars). Simulated data from the various models is superimposed in colour. Overall choice accuracy was higher in congruent conditions (i.e., go to reward and no-go to avoid loss) than incongruent conditions (i.e., go to avoid loss and no-go to reward), T(11) = 3.58, p = 0.004. Learning accuracy was higher for go versus no-go conditions, F(1,11) = 10.91, p = 0.007, and the interaction of action-by-valence was significant, F(1,11) = 12.80, p = 0.004. **C–F:** Learning curves showing average probability go over the course of 60 trials in each of the four conditions. The data is shown in black, and simulated data from the various models is superimposed in colour. **G:** Model comparison. Left panel shows how well the data is fitted in terms of average posterior choice probability. The right panel shows the integrated BIC in comparison to the best model. This penalizes models for complexity. The best model is the one with the lowest iBIC score. Here, the most complex model fits the data sufficiently well to warrant the complexity. **H–K:** Comparison of data and most parsimonious model (2 Pav) over the multiple sessions. Sessions are only concatenated for display purposes.

The effect size of BA (IDS-SR total score) was 3.46 Cohen’s d (large). See Tables 2, 3 and Figure 4A.

**Table 2:** Clinical course. IDS-SR = Inventory of Depressive Symptomatology. GAD 7 = Generalized Anxiety Disorder 7 item self-report. STAI (S/T) State Trait Anxiety Inventory.


VARIABLE	BASELINE	WEEK 4	WEEK 7	WEEK 10

IDS-SR	34.5 (8.5)	24.7 (9.7)	16.3 (7.9)	10.9 (6.3)*

GAD7	9.9 (3.9)	9.0 (5.3)	6.1 (5.5)	3.8 (3.1)

STAI State	43.9 (6.9)	41.8 (8.2)	41.1 (8.2)	35.3 (6.8)

STAI Trait	54.3 (7.6)	51.3 (8.0)	48.5 (10.4)	44.4 (11.5)*


**Table 3:** Response rates.


VARIABLE	DEFINITION	N (%)

Responder1	25% reduction IDS-SR score (baseline-Week 9)	12 (92.0%)

Responder2	50% reduction of the IDS-SR baseline to Week 9	10 (76.9%)

Remission	IDS below 14 at Week 9	8 (61.5%)


### 3.3 Behavioral measures of learning show specific influence of BA interventions

At baseline, the response pattern on the task was as previously reported (Figure 4B). Accuracy was higher in the congruent conditions (i.e., go to reward and no-go to avoid loss) than incongruent conditions (i.e., go to avoid loss and no-go to reward), T(11) = 3.58, p = 0.004. Learning accuracy was higher for go versus no-go conditions, F(1,11) = 10.91, p = 0.007, and the interaction of action-by-valence was significant, F(1,11) = 12.80, p = 0.004. All participants showed low performance in the nogo-to-win condition.

To establish the overall structure of decision-making, we first fitted a series of computational models to each session for each participant separately, assuming a single prior. Comparisons of real learning curves with those generated from the data for each task condition (Figure 4C-F) and formal Bayesian model comparison (Figure 4G) suggested the presence of previously established components, including standard Rescorla-Wagner instrumental learning, a bias towards active go responses and an irreducible noise component. In addition, there was evidence for two Pavlovian components. The first appetitive Pavlovian component *π*+ captured how appetitive expectations specifically promoted active go behavior, while the second aversive Pavlovian component *π–* captured how aversive loss expectations specifically inhibited active go behavior.

We next fitted a model with the aim of directly testing the main hypotheses. Our first hypothesis was that the appetitive interventions focusing on the pursuit of rewards would increase the appetitive Pavlovian component *π*+. The second hypothesis was that the aversive TRAP/TRAC intervention would reduce the aversive Pavlovian component *π–*. To test this, we built a novel computational model. Here, parameters were fixed for each subject across task administrations, except for *π*+ and *π*–. These two parameters were allowed to assume an initial value, and then to change to a different value for the remainder of the task administrations. The parameter *π*+ could assume a different value from task administration 3 onwards, and *π*– could assume a different value from task administrations 4 onwards. This model hence allowed us to measure a change in *π*+ due to the appetitive component of the intervention, and a change in *π–* due to the aversive component of the intervention. Figure 4H–K compares data generated from this model to the average learning trajectories over sessions, and reveals a satisfactory fit.

**Figure 5:**
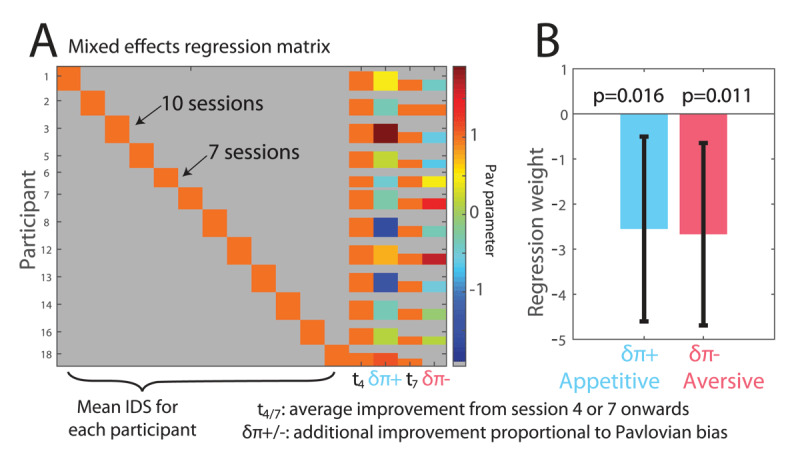
Relationship between therapeutic response and sustained task parameter changes. **A)** Mixed effects regression matrix. Temporal improvement in depression scores was modelled as a mixture of an individual mean, a fixed improvement after the appetitive (t3) and after the aversive (t7) session, and a fixed improvement proportional to the change in behaviourally measured positive and negative Pavlovian parameters after the appetitive and aversive intervention. **B)** Regression weights for both the positive and negative Pavlovian change parameters were significantly negative, suggesting that an increase in both appetitive and aversive Pavlovian parameters promoted symptom reduction.

We next asked whether the Pavlovian parameters related parametrically to the improvement in symptoms. To test this hypothesis, we built a mixed effects linear regression (Figure 5A). This model captured the time-course of total depression symptoms (IDS total scores; Figure 4A) as a weighted sum of five components including an average score for each individual; an average improvement from the appetitive intervention to the end and an average improvement from the aversive intervention to the end (orange components in the regression matrix in Figure 5A). In addition, it captured individual variability in improvement through two regressors proportional to each individual’s appetitive and aversive Pavlovian parameter. This revealed a (statistically significant) negative effect of each Pavlovian parameter change on the temporal evolution of the IDS scores (Figure 5B). Equivalent models allowing for changes in learning rates or irreducible noise parameters did not reveal a significant relationship to treatment response.

### 3.4 Changes in reward anticipation, punishment prediction and activity choices follow reinforcement-learning patterns

The results so far suggest that aspects of the improvement in depression symptom are related to changes in reinforcement learning. We next turned to the GOAL form to ask to what extent subjectively reported expectations about rewards would conform to formal reinforcement learning theories. In many algorithms from reinforcement learning, choices about what activity, *a*, to engage in are driven by respective estimates of how rewarding and punishing the activity will be, *R_a_* and *P_a_*. Typically *R_a_* and *P_a_* are learned from experience with activities and the respective rewards and punishments that they produce. For example, following engagement with an activity, *a*, a reward from the activity, *r*, is experienced, and a reward prediction error, *δ_R_*, is computed, as the difference between reward received and the reward expected at the time of choice, *δ_R_ = r–R_a_*. The expectation of future reward for activity *a*, is then incremented proportionally to the reward prediction error, Δ*R_a_* ∝ *δ_R_*, and this updated reward expectation is used to guide future choices. Analogous prediction error computations can be used to learn *P_a_* from experienced punishments.

**Figure 6:**
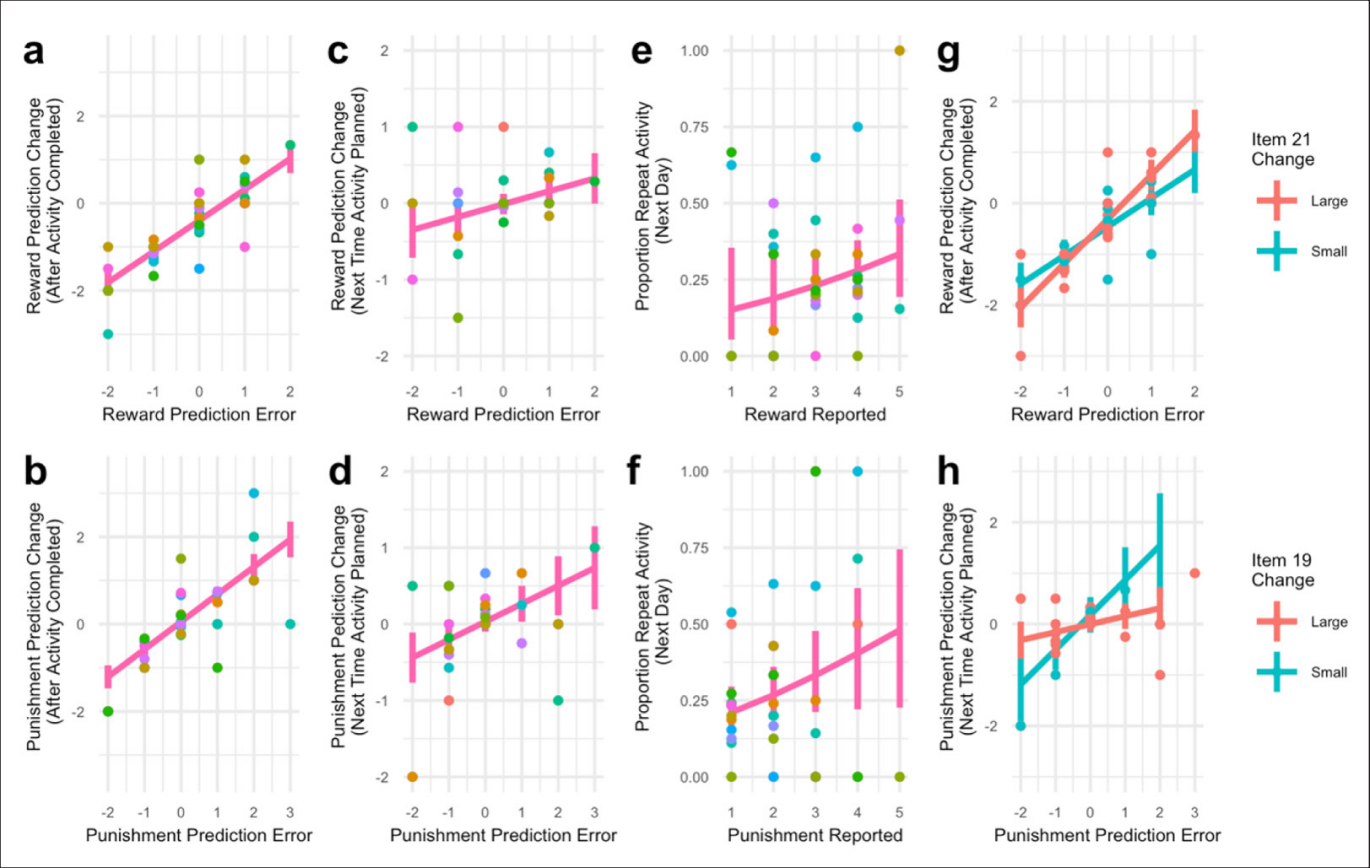
Reinforcement learning drives learning to evaluate activities and relates to improvements in anhedonia. **a)** Reward prediction errors predict changes in amount of reward predicted for engaging with an activity when reward prediction change is computed as the difference between reward predicted when the activity is planned and reward predicted immediately after it is completed. Reward prediction errors are defined as the difference between reward reported upon completing the activity and reward predicted when the activity was planned. **b)** Punishment prediction errors predict changes in amount of punishment predicted for engaging with an activity where punishment prediction change is computed as the difference between punishment predicted when the activity is planned and punishment predicted immediately after it is completed. Punishment prediction errors are defined as the difference between punishment reported upon completing the activity and punishment predicted when the activity was planned **c)** Reward prediction errors predict changes in amount of reward predicted for engaging with an activity when reward prediction change is computed as the difference between reward predictions for successive times the same activity is planned. **d)** Punishment prediction errors predict changes in amount of punishment predicted for engaging with an activity when punishment prediction change is computed as the difference between punishment predictions for successive times the same activity is planned. **e,f)** The chances of repeating the same activity two days in a row are modulated by both the reward (e) and punishment (f) reported on the first day. **g)** The effect of reward prediction errors on immediate reward prediction change is greater in individuals that have larger improvements on item 21, which measures capacity for pleasure or enjoyment, of the IDS-SR. **h)** The effect of punishment prediction errors on punishment prediction change between successive times the activity is planned lesser in individuals that have larger improvements on item 19, which measures general interest, of the IDS-SR. a-h) Points display averages of single subjects. For a-f each color corresponds to a different subject. For g-h, color corresponds to whether a subject’s item change was greater than the median. Lines show group-level predictions of mixed effects models. Error bars designate 95% intervals.

We examined whether subjects update estimates of the expected reward and punishment of activities using reward prediction errors, as suggested by reinforcement learning. To study this, prior to selecting an activity to perform for the following day, participants recorded a prediction of how rewarding and punishing they thought the activity would be, \[R_a^{pre}\] and \[P_a^{pre}\]. After performing the activity the following day, subjects recorded how rewarding and punishing they found the activity, *r*, and *p* and then made a prediction for how rewarding and punishing they would find the activity if they were to complete it again the next day, \[R_a^{(post1)}\] and \[P_a^{(post1)}\]. In order to examine whether these new predictions were driven by reward prediction errors, we computed reward and punishment prediction errors based on these responses, \[{\delta _{reward}} = r - R_a^{pre}\] and \[{\delta _{punishment}} = p - P_a^{pre}\], and examined whether these prediction errors explained change in reward and punishment predictions, \[\Delta R_a^1 = R_a^{(post1)} - R_a^{pre}\] and \[\Delta P_a^1 = P_a^{(post1)} - P_a^{pre}\]. Mixed effects linear regression revealed a significant effect of *δ_reward_* on \[\Delta R_a^1\] (Figure 6a, Estimate: 0.709, z = 10.53, p *<* 1e–25) as well as *δ_punishment_* on \[\Delta P_a^1\] (Figure 6b, Estimate: 0.634, z = 9.51, p *<* 1e–20), consistent with the hypothesis that subjects updated reward and punishment predictions using prediction errors.

We next investigated whether reward and punishment prediction errors explain changes in reward and punishment prediction over a longer time-scale. Previous research has suggested that while reward prediction error updating affects value updating over both short and long time-scale, the dynamics for each may be different (Eldar et al., 2018). In order to assess a longer time scale measure of prediction change, we examined instances where subjects re-planned to complete an activity that they had previously planned, possibly multiple days into the future, and recorded their reward and punishment predictions for this activity, \[R_a^{(post2)}\] and \[P_a^{(post2)}\]. We then, computed respective long-term measures of reward prediction change and punishment prediction change as the difference in reward predictions and punishment predictions for an activity, between successive times the activity was planned, \[\Delta R_a^2(a) = R_a^{(post2)} - R_a^{pre}\] and \[\Delta P_a^2(a) = P_a^{(post2)} - P_a^{pre}\]. These measures were also affected by *δ_reward_* (Figure 6c, Estimate = 0.168, z= 2.07, p = .038) and *δ_punishment_* (Figure 6d, Estimate = 0.228, z = 2.04, p = .0416) respectively, suggesting that reward and punishment prediction errors can affect changes in reward and punishment predictions over a longer time-scale.

We next investigated whether reward and punishment predictions, learned through prediction errors, are used to guide choices about what activity to engage in. A result of this process is that activities should be chosen based on their recently observed rewards and punishments. A commonly used analysis to assess the presence of this result is to examine the odds of repeating an activity from a previous day as a function of the reward that it recently generated (Lau and Glimcher, 2005). We observed that the chances of repeating an activity from the previous day were positively affected by the reward that that activity produced (Figure 6; Estimate = 0.32, z = 2.10, p = .044) providing evidence that reward estimates, learned by prediction errors are used to guide choices about what activities to engage in. Surprisingly, we also observed that the chances an activity was repeated were also positively effected by reported punishment, perhaps reflecting a focus in therapy to continue to engage with difficult activates (Estimate = 0.45, z = 2.81, p = .005).

Next, we were interested in whether the efficacy of learning processes relates to improvement in anhedonia symptoms. To examine this, we isolated two questions from the IDS-SR relating to improvements in anhedonia: item 19 which measures general interest, and item 21 which measures capacity for pleasure or enjoyment. We found the effects of *δ_R_* on Δ*R*_1_ were greater in individuals that had greater changes in item 21 over the course of the therapy (Figure 6g. Estimate = 0.22, z = 2.67, p_uncorrected_ = .010, p_corrected_ = .020), providing some evidence that the efficacy of reinforcement learning processes drive improvements in anhedonia. We did not find differences in item 19 (Estimate = 0.173, z = 1.07, p = .284). Additionally, improvements in either item were not related to effects of *δ_R_* on Δ*R*_2_ (item 19: Estimate = –0.27, z = –0.95, p = 0.341, item 21: Estimate = .061, z = .47, p = .640).

With regards to punishment predictions, we found that the effects *δ_P_* on Δ*P*_2_ were lesser in individuals that had greater changes in item 19 over the course of the therapy, although this effect narrowly did not survive multiple comparisons correction (Figure 6h. Estimate = –0.541, z = –2.16, p_uncorrected_ = .031, p_corrected_ = .062). We did not find differences in item 21 (Estimate = –0.189, z = –1.50, p = .134). Additionally, neither item was related to effects of *δ_P_* on Δ*P*_1_ (Item 19: Estimate = –0.068, z = –0.36, p = .717; Item 21: Estimate = –0.190, z = –0.50, p_uncorrected_ = .620).

For both significant interactions with IDS-SR questions, we examined whether these could be accounted for by baseline IDS-SR scores. Repeating these analysis yet replacing change in the questionnaire item with its baseline value, revealed that individuals higher in item 21 at baseline demonstrated greater effects of *δ_R_* on Δ*R*_1_ (Estimate = –.221, z = –2.44, p_uncorrected_ = .015, p_corrected_ = .030). The same analysis was not significant when examining the effect of baseline scores in item 19 on the effect of *δ_P_* on Δ*P*_2_ (Estimate = –.167, z = –1.16, p_uncorrected_ = .244). This suggests a possibility that modulation of the effect of *δ_R_* on Δ*R*_1_ by change in item 21 scores could be accounted for by baseline item 21 scores. Disentangling this will require a larger sample.

Finally, we asked whether the learning rates implied by task choices and the GOAL form were related. However, there were no significant correlations.

## 4 Discussion

This pilot study examined whether formal reinforcement learning theories could potentially account for the response to behavioral activation therapy for depression. This contention was supported in several ways. First, as hypothesized, we found that the response to the component of BA which emphasizes active engagement with reward resulted in an increased appetitive Pavlovian effect on choice in those individuals who responded to that part of the therapy. We also found a relationship between changes in aversive Pavlovian effects on choice, but this did not go in the hypothesized direction. Third, we found that self-reported reinforcer experience and anticipation changes follow formal RL rules, and that individual differences in this relate to differences in treatment response. Hence, the results of this study provide initial support for the suggestion that response to BA may be mediated via RL mechanisms, and that individual differences in these RL mechanisms may account for some of the individual differences in the response to BA treatment.

Beyond the fact that RL mechanisms showed a relationship to improvement, probably the most tantalizing finding is the suggestion that different components of BA may engage different neurocognitive components – with reward pursuit relating to Pavlovian approach and the reduction of unhealthy avoidance relating to Pavlovian inhibition. The ability to identify such specific effects was facilitated by the BA treatment manual employed, which represented an adapted version of standard treatment manual. The aim of the adaptation was to better separate specific components of the therapy such that their specific effects in terms of task changes could be better distinguished. An ability to identify components of psychotherapies and relating them to specific neurocognitive mechanisms may be useful both in tailoring therapies to individuals, and in prediction treatment response to specific components. The specific relationship identified was as anticipated in terms of appetitive Pavlovian processes. However, in the case of aversive Pavlovian processes, the effect went against our expectations. During the second phase of the therapy, a TRAP/TRAC procedure was employed. Here, participants identify triggers (T), their usual emotional response (R) and avoidance pattern (AP), and attempt to formulate an alternative, more adaptive active coping (AC) approach. We had anticipated that this would facilitate the maintenance of active behavior in the face of aversive expectations, and hence reduce Pavlovian aversive inhibition, but found the opposite. One possibility is that the final therapy input, the learning of avoiding maladaptive behaviors may have influenced this, but there were not sufficient data to clearly examine this. A second possibility is that the process of TRAP/TRAC might itself depend on a type of inhibition, namely of the habitual avoidance pattern. It is hence tempting to speculate that successful engagement in TRAP/TRAC promoted Pavlovian inhibition in the sense that it promoted a general inhibition for reflective purposes. We note here that exploratory analyses with models which allowed for separate learning rates for rewards or losses, or separate irreducible noise terms, changes in learning rates or irreducible noise parameters did not show significant relationships to treatment response, providing suggestive evidence that the observed treatment effects may be specific to Pavlovian processes.

The second finding of note is that individuals who updated their expectations of rewards more according to reward prediction errors showed a greater improvement in anhedonic symptoms. It chimes well with recent reports whereby anhedonia involves an impairment in precisely this kind of neural updating (Greenberg et al., 2015; 2019; Eckstrand et al., 2019). Interestingly, behavioral RL work has shown some support both for the notion that anhedonia relates to a reduction in reward (Huys et al., 2013; Steele et al., 2007; Chase et al., 2010; Kunisato et al., 2012; Blanco et al., 2013; Robinson and Chase, 2017) or punishment sensitivity (Beevers et al., 2013; Herzallah et al., 2013), and that it relates to the process of learning from rewards itself (Chase et al., 2010). However, what had not been examined yet is what accounts for changes in anhedonia. The results presented here suggest that the process of learning may relate to *changes* in anhedonic symptoms through therapy. A similar qualitative interpretation is supported by a recent larger study examining reinforcement learning changes over the course of cognitive behaviour therapy for depression: Brown et al. 2021. This study examined a simpler paradigm requiring participant only to learn which of two stimuli led to more rewards or less losses. They found that reward sensitivity and learning rate were associated with depression at baseline, and improved during therapy. Our results extend their findings by examining Pavlovian processes in a more specific manner, and by showing similar reinforcement learning processes (though apparently on different timescales) also govern self-reported expectations and their changes.

Finally, we note that the GOAL form was designed to measure changes in the experience and anticipation associated with activity types, but was not designed to measure changes in the number or frequency of such activities. Such measures are often used as proximal evidence that behavioural interventions had the desired effect. However, here we are aiming to examine a more proximal and subtle measure of the effect of interventions. As this is hypothesized to lie upstream of the scheduled activities (by altering decision processes which lead to activities), it is not dependent or predicated on a change in the number of type of activities scheduled.

### Limitations

We acknowledge important limitations. Most importantly, the sample in this pilot study size is very small, and this is particularly critical as we examine individual differences in treatment response. As such, the results presented here require replication in larger samples. We note in particular that the nogo to avoid learning was not captured very well by the model, suggesting that other cognitive processes might be at play which we have not appropriately measured. The small sample size unfortunately precludes meaningful model development to understand this fully. Nevertheless, the analyses rely on extensive sampling of individuals, both with multiple task administrations and with multiple GOAL form evaluations. In addition, the treatment intervention timing was not randomized, which may have given rise to order effects. As such, it is possible that the change in the aversive Pavlovian parameter observed here may not be driven solely by the therapy focus on reducing unhealthy avoidance and rewards, but may depend on the early interventions. In order to disentangle this, it will be necessary to either examine therapy components separately, or to randomize the order of components during therapy. Finally, we note the presence of comorbid disorders. The small sample size again precludes analyses to clarify their impact.

## 5 Conclusions

In a small pilot study, individual differences in responding to behavioral activation therapy for depression were longitudinally associated with multiple facets of reinforcement learning. These data suggest that behavioral activation may engage reinforcement learning mechanisms, and that treatment response may be moderated by individual differences in these mechanisms.

## Additional File

The additional file for this article can be found as follows:

10.5334/cpsy.81.s1 Appendix. Daily GOAL Check.
