# Supplementary material for: Components of Behavioral Activation Therapy for Depression Engage Specific Reinforcement Learning Mechanisms in a Pilot Study
Source: Comput Psychiatr. 2022 Oct 13;6(1):238–55. doi: 10.5334/cpsy.81 (PMC11104310; doi:10.5334/cpsy.81)
Supplement: Appendix. — Daily GOAL Check. [file cpsy-6-1-81-s1.pdf]

APPENDIX

NU STU#00206862: Go/No-Go Learning Study
Subject ID: ____ Date of completion_____________________
Follow-up day____

Daily GOAL Check

Instructions: What is your planned goal for tomorrow? How did your planned goal make you feel today? Circle the number that best reflects your experience.

A. Your Current Feelings

1. Right now, do you feel interested, excited, strong, enthusiastic, strong, proud, alert, inspired, attentive, determined, active?
How positive do you feel? (Circle one number below) *[Variable: Positive emotional state, PANAS_P]*
   - 1 = Very slightly or not at all
   - 2 = A little
   - 3 = Moderately
   - 4 = Quite a bit
   - 5 = Extremely

2. Right now, do you feel irritable, guilty, distressed, upset, anxious, scared, hostile, ashamed, nervous, jittery, afraid?
How negative do you feel? (Circle one number below). *[Variable: Negative emotional state, PANAS_N]*
   - 1 = Very slightly or not at all
   - 2 = A little
   - 3 = Moderately
   - 4 = Quite a bit
   - 5 = Extremely

Subject ID: ____ Date of completion_____________________
Follow-up day_______________________________________

B. Tomorrow's Planned Activity

*[Reinforcement learning with reward, New expectation = old expectation + learning rate * (reinforcement – old expectation)]*

3. Circle one activity that you plan for tomorrow that will improve your symptoms. *[Variable: tomorrow anchor]*
    1. Emotional activities that aim to accept or experience your feelings
    2. Mental activities that challenge yourself to think about new ideas
    3. Physical activities that improve your physical health
    4. Pleasure activities that increase your joy or delight
    5. Sensory activities that increase your sight, smell, sound, taste, or touch
    6. Social activities connecting with others
    7. Spiritual activities that show your values

Describe the planned activity? ____.

How certain are you that you will do this activity?" I am _____ percent (%) certain that I will do this activity.

4. To what extent will this planned activity make you feel positive tomorrow? *[Variable: old positive expectation/value]*
    1 = Very slightly or not at all
    2 = A little
    3 = Moderately
    4 = Quite a bit
    5 = Extremely

5. Will you automatically want to do this activity tomorrow because you predict it will be pleasant? *[Variable:anticipated Pavlovian approach]*
    1 = Very slightly or not at all
    2 = A little
    3 = Moderately
    4 = Quite a bit
    5 = Extremely

6. Will this planned activity make you feel negative tomorrow? *[Variable: old negative expectation/value]*
    1 = Very slightly or not at all
    2 = A little
    3 = Moderately
    4 = Quite a bit
    5 = Extremely

7. Will you automatically want to avoid today's activity because you predict it will be UNpleasant? *[Variable: anticipated Pavlovian avoidance]*
    1 = Very slightly or not at all
    2 = A little
    3 = Moderately
    4 = Quite a bit
    5 = Extremely

C. Today's Activity

8. Circle one activity that you did <u>today</u> that improved your symptoms. *[Variable: today anchor]*
    1. Emotional activities that aimed to accept or experience your feelings
    2. Mental activities that challenged yourself to think about new ideas
    3. Physical activities that improved your physical health
    4. Pleasure activities that increased your joy or delight
    5. Sensory activities that increased your sight, smell, sound, taste, or touch
    6. Social activities connected with others
    7. Spiritual activities that showed your values

Describe the activity you did today? ____.

9. Was this yesterday's planned activity?

    1 = yes
    2=no

10. Did today's activity make you feel positive?

    1 = Very slightly or not at all *[Variable: reward]*
    2 = A little
    3 = Moderately
    4 = Quite a bit
    5 = Extremely

11. Did today's activity make you feel negative? *[Variable: punishment]*

    1 = Very slightly or not at all
    2 = A little
    3 = Moderately
    4 = Quite a bit
    5 = Extremely

12. Did you automatically want to approach today's activity because it was pleasant?

    1 = Very slightly or not at all *[Variable: experienced Pavlovian approach]*
    2 = A little
    3 = Moderately
    4 = Quite a bit
    5 = Extremely

13. Did you automatically want to avoid today's activity because it was <u>un</u>pleasant?

    1 = Very slightly or not at all *[Variable: experienced Pavlovian inhibition]*
    2 = A little
    3 = Moderately
    4 = Quite a bit
    5 = Extremely

14. If today's activity was unpleasant, did you have to override or push past your automatic avoidance?
1 = Very slightly or not at all *[Variable: Pavlovian inhibition override effort]*
2 = A little
3 = Moderately
4 = Quite a bit
5 = Extremely

15. If you did this activity tomorrow, how pleasant would it be?
1 = Very slightly or not at all *[Variable: new positive expectation]*
2 = A little
3 = Moderately
4 = Quite a bit
5 = Extremely

16. If you did this activity tomorrow, how UNpleasant would it be?
1 = Very slightly or not at all *[Variable: new negative expectation]*
2 = A little
3 = Moderately
4 = Quite a bit
5 = Extremely

17. Would you plan today's activity for tomorrow because you would have a rewarding experience?
1 = Very slightly or not at all *[Variable: positive reinforcement]*
2 = A little
3 = Moderately
4 = Quite a bit
5 = Extremely

18. Would you plan today's activity for tomorrow because you would avoid a negative experience?
1 = Very slightly or not at all *[Variable: negative reinforcement]*
2 = A little
3 = Moderately
4 = Quite a bit
5 = Extremely
